# Numerous Paragraphs of General Interest to the Profession

**Published:** 1893-07

**Authors:** 


### Medical News and Miscellany.

---

**Dr. L. M. Stroud** has removed from Forney to Terrell, Texas.

---

**Died,** at New Boston, Texas, May 29, 1893, Dr. J. M. Ball, an old resident physician of Bowie county.

---

**To be** "in the swim" is now expressed—"participating in the aquatic gymnastics of the natatorium."

---

**Dr. Sam Cunningham,** of Elgin, is taking a course at the New York Polyclinic—a great favorite with Texas physicians.

---

**Prof. A. J. Smith's** "copy" for his department (Medicine) and his paper on Rail Road Spine, came too late for this issue.

---

**Dr. L. L. Shropshire,** of San Antonio, is at the New York Polyclinic; will leave there July 15th for Chicago, and be at home August 1st.

---

**A Sudden Death.**—While riding on a horse, Dr. Farrell, of Gregory, fell from the saddle, and remained in an unconscious condition until he died, June 22.

---

**Married.**—In Monterey, Mexico, June — ult., Miss Lee Boyers to Dr. George Stell, both of Monterey. Dr. Stell is a son of Dr. W. W. Stell, of Monterey, late of Austin, Texas.

---

**Dr. A. C. Walker,** long time resident of Rockdale, has, a second time, removed to Fort Worth, and joined Dr. E. J. Beall in the general practice. They do an immense amount of surgery.

---

**Dr. W. C. Fisher,** whose paper appears in this issue, has been appointed City Physician of Galveston, with a good salary. This appointment was one of the first official acts of His Honor, Mayor (Dr.) A. W. Fly. *Good.*

**Married.**—At Orange, Texas, June 27th ult., Miss Laura, daughter of Mr. and Mrs. A. Gilmer, to Dr. Frederick Hadra, both of Orange, Texas. Dr. Hadra is the son of Dr. B. E. Hadra, the distinguished surgeon and gynecologist.

---

**Government Medical School.**—Secretary Lamont has adopted the recommendation of Surgeon General Sternberg, and ordered the establishment of an army medical school, in Washington, for instruction of approved candidates for admission to the medical corps of the army.

---

A Thoroughly Competent Druggist, twenty-eight years of age, married, desires a situation. Speaks Spanish and English, and has had ten years experience. Best testimonials as to character, and habits and qualifications. Address "D," care this journal, Austin, Texas.

---

**Dr. West's Letter** in our June number about the *Buffet Banquet* at the Galveston meeting has been sharply criticised, and the JOURNAL is in receipt of a letter upon this subject from a prominent member, an ex-President, which came too late for this issue. It will appear in our next.

---

**Dr. A. W. Fly** was elected Mayor of Galveston, June 1st, by a tremendous majority, defeating Col. Fulton by 1893 votes, out of 3000 odd. Fulton had held the office ten consecutive years. Fly is a *trump* and will make a lightning good Mayor, or a good anything where brains and pluck are the requisites.

---

**This,** the initial number of TEXAS MEDICAL JOURNAL, known for eight years as "*Daniel's* Texas Medical Journal," and the initial number of Vol. 9, enlarged and improved, is *decidedly* a medical college number, our list of medical college advertisements embracing nearly every college of any repute. See them all.

---

**There** are so many graduates of the Memphis Hospital Medical College in Texas, that they are organized, and hold annual reunions. The reunion of 1893 was held at Dallas, June 20th ult., and there was a large attendance. Why not let the *Alumni* of other colleges organize also? At some schools besides Memphis, there are from fifty to seventy-five Texas students each year.

**Dr. O. M. Turner,** of Mississippi, a graduate of Tulane University Medical College, and a son of Dr. D. B. Turner, of Winona, Miss., has located in Austin to practice. Dr. Turner brings letters of endorsement from leading Mississippi physicians, and we bespeak for him a courteous reception by the physicians of Texas.

---

**Dr. P. K. Wortham** has removed from Waco to Reisel, McLennan county, Texas. Dr. Wortham, it will be remembered, suffered heavy loss by fire, not long ago, in Waco, having his house and contents destroyed. The JOURNAL extends best wishes to Dr. Wortham, and hopes that in his new home he may speedily recoup his losses.

---

**Drs. Ralph Steiner and J. O. Lewright,** of Austin, will shortly sail for Europe. They go to Germany to study. Dr. Steiner will remain two years, fitting himself for a specialty—chest and throat—and will return to Austin. Dr. Lewright takes a general course. They will correspond with the JOURNAL, giving all the clinical news of importance.

---

**For Sale.**—Dr. W. B. Anderson, whose card appeared in the JOURNAL last winter, now, since his expected trip is close at hand, offers his property for less than cost. This is certainly an unusual opportunity, for the Doctor can put a physician into an annual $2000 practice at once without opposition. Address him at CONTENT, RUNNELS CO., TEXAS.

---

**A sensation** was created in Austin recently by the death of a well known dipsomaniac while taking the "treatment" at the "Hagey Institute." Old Capt. Orr was the patient. The Hagey people say that he violated his pledge to not drink elsewhere than at the "institute," went off and got terribly drunk, and—died. No inquest was held.

Tally *two* in Austin; one laid to the homeopathic Keely, and this one to its rival, the Hagey.

---

**Death of Dr. Becton's Daughter.**—The JOURNAL learns with much regret of the death of Mrs. Mary O. Chandler, daughter of Dr. E. P. Becton, of Sulphur Springs. She died at her father's house, May 31. Mrs. Chandler was born at Tarrant, Hopkins county, Texas, August the 8th, 1872. She was educated at Central College, graduating from that institution in letters and music with the highest honors of the class in 1890. She was married to Rev. S. E. Chandler, Professor of Systematic Theology and Greek in Austin College, Sherman, December 2d, 1892. She was laid to rest in the city cemetery in Sulphur Springs, June 1st ult. The JOURNAL tenders its sincere sympathy to the bereaved family.

---

**Death of Dr. Powell.**—Dr. Belitha Powell, one of the oldest resident physicians of Houston, was found dead in bed the morning of June 17th ult., supposed to have died of heart disease. Dr. Powell was born in Maryland, May 3, 1832. He was a graduate of Jefferson Medical College of the class of 1853. He was chief surgeon of the 4th Louisiana battalion, in the Confederate Army, during the early part of the war; was transferred to the Trans-Mississippi Department in 1862, and organized the hospitals of that department. Settled in Houston in 1866, and resided there continuously till date of his death. His wife, to whom he was married in September, 1857, was Sallie G. Harvey, of Madison parish, Louisana.

---

**Run the Blockade.**—A death from small-pox occurred recently in Torreno, Mexico. The man's wife took her child, and passing through Eagle Pass, went to San Antonio. The child there developed small-pox. This was promptly reported to State Health Officer Swearingen, who at once instituted inquiry as to how the woman got into Texas, and ascertained that she swore she had not been in contact with small-pox, nothwithstanding she was just from the room in which her husband died,—thus not only violating the quarantine laws of the State, but committing perjury. The case is being further investigated; meantime Quarantine Inspector Lott, of Eagle Pass, is fully exonorated from blame. No other cases occurred.

---

**Surgical Instruments.**—A recent number of Meyer Bros. & Co.'s St. Louis *Drug Magazine* states that the Fort Worth Pharmacy Company, of Fort Worth, carries the largest stock of Surgical Instruments and mechanical curative devices in the Southwest. We are personally acquainted with these people and have a correct knowledge of their stock, fully endorse the above, and will further say, their prices we know to be as low as any of the Eastern houses, and that they are now giving good satisfaction to five hundred Texas doctors whom they number as their patrons. A year ago we asked the profession to aid us in building up this house—a home institution—where orders could be promptly filled and delivered. We have now to say that satisfactory results are being obtained. The Fort Worth Pharmacy Company are also agents for three manufactories of Electric Batteries and two manufactories of Physicians' Chairs. Write to them for what you may want.

